# Remnant Preservation in Anterior Cruciate Ligament Reconstruction Versus Non‐Preservation Methods: A Systematic Review and Meta‐Analysis

**DOI:** 10.1111/os.70167

**Published:** 2025-09-04

**Authors:** Chao Fan Chen, Ling Yao Kong, Tao Li, Lei Yao, Yang Xu, Li Wang, Hong Yu Zhou, Jian Li

**Affiliations:** ^1^ Sports Medicine Center, West China Hospital, Sichuan University, Department of Orthopedics and Orthopedic Research Institute West China Hospital, Sichuan University Chengdu China; ^2^ Department of Neurology West China Hospital, Sichuan University Chengdu China

**Keywords:** anterior cruciate ligament, anterior cruciate ligament reconstruction, meta‐analysis, remnant preservation

## Abstract

To manage anterior cruciate ligament (ACL) injury, both remnant‐preserving anterior cruciate ligament reconstruction (ACLR) and standard ACLR without remnant preservation are applied. This study aims to systematically evaluate clinical outcomes of remnant‐preserving versus standard ACLR techniques by analyzing randomized controlled trials (RCTs). The PubMed, Embase, and Cochrane Library databases were used to identify studies published from January 2000 to November 2024. Based on the PICOS framework, we systematically reviewed RCTs in which patients with ACL injuries compared ACLR with remnant preservation versus standard ACLR in terms of International Knee Documentation Committee (IKDC) score, Lysholm score, Lachman test, pivot shift test, KT1000/2000 arthrometer side‐to‐side difference (SSD), synovial coverage, proprioception evaluation, cyclops lesion, and range of motion (ROM). Data were pooled using the random‐effects model or fixed‐effects model, based on the heterogeneity. The quality of the included literature was assessed based on the Cochrane Risk of Bias tool (ROB 2.0), and the GRADE criteria were applied to rate evidence quality for key outcomes. Review Manager 5.4 and Stata 15 were used for the statistical analyses. The relative risk (RR) was used for dichotomous data, and the mean difference (MD) was used for continuous variable data. Both types of indicators were expressed as 95% confidence intervals (CIs). The minimal clinically important difference (MCID) was adopted to determine whether patients improved enough clinically to notice a difference. Subgroup analyses were conducted for outcomes failing to reach MCID thresholds in order to examine potential modifiers of different follow‐up durations and remnant preservation techniques. A total of 10 studies were included in the qualitative review and meta‐analysis. Although there were statistically significant differences between the remnant preservation group and the standard technique group in favor of the remnant preservation technique with respect to postoperative Lysholm score (MD 1.44; 95% CI, 0.60–2.29; *I* ^2^ = 23%; *p* < 0.01) (GRADE: Moderate), SSD (MD −0.57; 95% CI, −0.98 to −0.15; *I* ^2^ = 86%; *p* < 0.01) (GRADE: Low) and proprioception recovery (MD −0.57; 95% CI, −0.83 to −0.31; *I* ^2^ = 0%; *p* < 0.01) (GRADE: Low), these observed differences are so small that they are unlikely to be clinically relevant. No differences were found in other clinical outcomes between the two groups. The follow‐up duration and remnant preservation techniques were not identified as the key factors influencing the differences between remnant preservation ACLR and standard ACLR. No clinically meaningful benefit in postoperative knee stability or function; remnant preservation may be considered primarily when technical feasibility is high and remnant quality is optimal. This is achieved without increasing the risk of cyclops lesions and deficiency of ROM.

## Graphical abstract

To manage anterior cruciate ligament (ACL) injury, both remnant‐preserving anterior cruciate ligament reconstruction (ACLR) and standard ACLR without remnant preservation are applied. This study aims to systematically evaluate clinical outcomes of remnant‐preserving versus standard ACLR techniques by analyzing randomized controlled trials (RCTs). We systematically reviewed RCTs in which patients with ACL injuries compared ACLR with remnant preservation versus standard ACLR, in terms of International Knee Documentation Committee (IKDC) score, Lysholm score, Lachman test, pivot shift test, KT1000/2000 arthrometer side‐to‐side difference (SSD), synovial coverage, proprioception evaluation, cyclops lesion, and range of motion (ROM). Data were pooled using the random‐effects model or fixed ‐effects model, based on the heterogeneity. The quality of the included literature was assessed based on the Cochrane Risk of Bias tool (ROB 2.0), and the GRADE criteria were applied to rate evidence quality for key outcomes. A total of 10 studies were included in the qualitative review and meta‐analysis. Although there were statistically significant differences between the remnant preservation group and the standard technique group in favor of the remnant preservation technique with respect to postoperative Lysholm score, SSD, and proprioception recovery, these observed differences are so small that they are unlikely to be clinically relevant. No differences were found in other clinical outcomes between the two groups. Given moderate‐certainty evidence showing no clinically meaningful benefit in postoperative knee stability or function, remnant preservation may be considered primarily when technical feasibility is high and remnant quality is optimal.

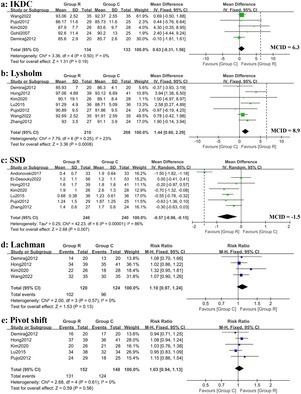

**Abbreviations**
ACL: anterior cruciate ligament
ACLR: anterior cruciate ligament reconstruction
CI: confidence interval
IKDC: international knee documentation committee
MCID: minimal clinically important difference
MD: mean difference
RCT: randomized controlled trial
ROM: range of motion
RR: relative risk
SSD: KT1000/2000 arthrometer side‐to‐side difference

## 1 Introduction

Injuries of the anterior cruciate ligament (ACL) occur in approximately 50% of all knee‐related injuries requiring surgery, with an annual incidence of 46/100,000 [1, 2]. ACL injury may result in recurrent knee instability, decreased activity, meniscal injuries, and increased risk of knee osteoarthritis [3]. Arthroscopic reconstruction of the ACL (ACLR) has become the current treatment for patients with severe symptoms or who attempt to return to high‐intensity sports [4, 5, 6, 7].

Nowadays, the importance of remnant tissue during ACL reconstruction has been increasingly recognized. Theoretically, remnant preservation allows native tissue to grow into the graft to increase mechanoreceptors in the grafted tendon [8, 9]. Other potential advantages include the creation of a watertight seal to prevent tibial tunnel widening and more accurate graft positioning [8, 10]. However, this method is argued in terms of an increased risk of impingement and cyclops lesions [11, 12].

Recent studies reported that, in comparison with standard ACLR without remnant preservation, the ACL remnant‐preserving technique provides better knee stability and proprioceptive function, and patients returned to sports activities early and achieved their previous performance levels efficiently [13, 14, 15, 16]. However, several studies have suggested that the remnant‐preserving technique does not enhance patient‐reported scores or knee stability. Instead, this approach increases the complexity of the procedure, resulting in increased complications [17, 18, 19, 20, 21].

Despite the conclusion of recent systematic reviews that remnant preservation has some advantages over standard techniques, some studies have pooled randomized controlled clinical trials (RCTs) with cohort studies for comparison and have not performed subgroup analyses [22, 23, 24]. To address these limitations, this review analyzed RCTs only, minimizing confounding bias and enabling robust comparisons between remnant‐preserving and standard ACLR techniques in terms of (1) primary outcomes (Lachman test, pivot‐shift test, and KT‐1000/2000 arthrometer side‐to‐side difference, International Knee Documentation Committee objective score and Lysholm score) and (2) secondary outcomes (synovial coverage, proprioception evaluation, cyclops lesion and range of motion).

## 2 Methods

This systematic review and meta‐analysis was performed and reported in line with the Preferred Reporting Items for Systematic Reviews and Meta‐Analysis (PRISMA) Statement [25]. The protocol was prospectively registered with a prespecified methodology on PROSPERO (CRD420251036969).

### 2.1 Literature Search

Five authors independently and systematically searched three databases (PubMed, Embase, Cochrane Library). Key terms used to conduct the search were selected and combined with the following English terms and their equivalents: anterior cruciate ligament AND (remnant OR stump OR residual) (See Appendix A–C). This search was limited to studies reporting outcomes in the last 24 years (from January 2000 to November 2024) and was written in English. According to the inclusion and exclusion criteria, five authors independently selected all articles by reviewing the full text. Any disagreements at the inclusion stage were resolved by discussion with the corresponding author.

### 2.2 Inclusion and Exclusion Criteria

The inclusion criteria for this article were as follows: (1) RCTs published in English; (2) ACL reconstruction with remnant preservation performed on the experimental group (Group R), whereas standard ACL reconstruction without remnant protection was performed on the control group (Group C), and the surgical techniques were fully described; and (3) at least one of the following outcome measures was reported: postoperative International Knee Documentation Committee (IKDC) score, Lysholm score, Lachman test, pivot shift test, KT1000/2000 arthrometer side‐to‐side difference (SSD), synovial coverage, proprioception evaluation, cyclops lesion, range of motion (ROM).

The exclusion criteria were as follows: (1) non‐RCTs (cohort studies, case control studies, case reports, review articles, animal or cadaver studies); (2) literature published repeatedly; (3) full text that could not be obtained; (4) studies that did not directly compare the outcomes of remnant‐preserving ACLR and standard ACLR; and (5) studies in which patients had fractures on the same side or previous knee surgery (except diagnostic arthroscopy or meniscus surgery).

### 2.3 Literature Screening

We imported all the retrieved literature into Endnote and then excluded duplicate literature. After reading the title or abstract, we filtered out preliminary studies and download the full text. We read the full text and screened out the original studies that fitted this systematic review. According to the inclusion and exclusion criteria, three authors independently selected all articles and cross‐checked them by reviewing the full text. Any disagreements at the inclusion stage were resolved by discussion with the corresponding author.

### 2.4 Data Extraction

All the data were extracted using a standardized extraction spreadsheet that included title, first author, year of publication, treatment method, sample size, mean age, sex ratio of patients, time from injury to surgery, duration of follow‐up, and clinical outcomes. The primary outcomes included postoperative IKDC score, Lysholm score, KT1000/2000 arthrometer side‐to‐side difference, Lachman test, and pivot shift test. The second outcomes included synovial coverage, proprioception evaluation, cyclops lesion, and range of motion. The proprioceptive function was evaluated by the passive angle reproduction test. In this test, patients were seated on the Biodex detector, wearing a blindfold and earplugs, and their knees were kept in a neutral rotational position. The affected knee was first flexed to 90°, and then passively extended gradually with an angular velocity of 5°/s by the Biodex detector, stopping at the set angle. Following a few repetitions to ensure the patient's familiarity with the angle, the affected knee is permitted to return to a flexion of 90°. Upon the patient's perception of the knee's angle reaching the pre‐established setting, he or she would press the stop button. The angle would then be documented, and the discrepancy from the set angle would be calculated.

### 2.5 Quality Assessment

The quality of the included literature was assessed by five researchers based on the Cochrane Risk of Bias tool (ROB 2.0). The literature will be categorized as “low risk,” “high risk,” “unclear risk,” and any discrepancies in the assessment will be judged after discussion with the corresponding author. Reviewers assessed articles for bias in six areas: random sequence generation, concealment of treatment allocation, blinding of participants and personnel, blinding of outcome assessors, incomplete outcome data, selective reporting, and other bias.

### 2.6 Evidence Certainty Evaluation

Two independent reviewers applied GRADE criteria to rate evidence quality (High/Moderate/Low/Very Low) for key outcomes, assessing five domains: risk of bias, inconsistency, indirectness, imprecision, and publication bias [26]. Discrepancies were resolved through panel discussion.

### 2.7 Clinical Significance Thresholds

In addition to statistical significance when interpreting and reporting investigation results, the minimal clinically important difference (MCID) is a useful benchmark to determine whether patients improve enough clinically to notice a difference [27]. Therefore, we adopted the validated Lysholm score MCID of ≥ 8.9 points, IKDC score MCID of ≥ 6.3 points, and KT‐1000 side‐to‐side difference threshold of ≥ 1.5 mm per international consensus guidelines [28, 29].

### 2.8 Statistical Analysis

Review Manager 5.4 and Stata 15 were used for the statistical analyses. The relative risk (RR) was used for dichotomous data, and the mean difference (MD) was used for continuous variable data. Both types of indicators were expressed as 95% confidence intervals (CIs). For continuous outcomes, we calculated the ratio of mean difference to MCID (MD/MCID%). Effects below 50% of MCID thresholds were flagged as “clinically equivocal” regardless of statistical significance. Regarding the heterogeneity test, the studies with good homogeneity (*I* ^2^ ≤ 50%) were used the fixed‐effects model for the meta‐analysis. If the studies had high heterogeneity (*I* ^2^ > 50%), the random‐effects models were used for the meta‐analysis, and the sensitivity analyses were conducted utilizing the sequential elimination method. In this method, the documents were systematically excluded one by one, with the remaining documents then being included for the meta‐analysis. The impact of specific studies on the results of the meta‐analysis was then assessed by examining the alterations in the merged results. Funnel plots were used to check the publication bias of the involved articles. To assess whether the treatment effect varied with time and remnant preservation techniques, as longer follow‐up might be needed to observe clinically meaningful changes in patients with ACLR, we conducted exploratory subgroup analyses for outcomes failing to reach MCID thresholds, examining potential modifiers of different follow‐up duration (≤ 12 months and ≥ 24 months) and remnant preservation techniques (tensioning or suturing and simple preservation).

## 3 Results

### 3.1 Literature Search

**FIGURE 1:**
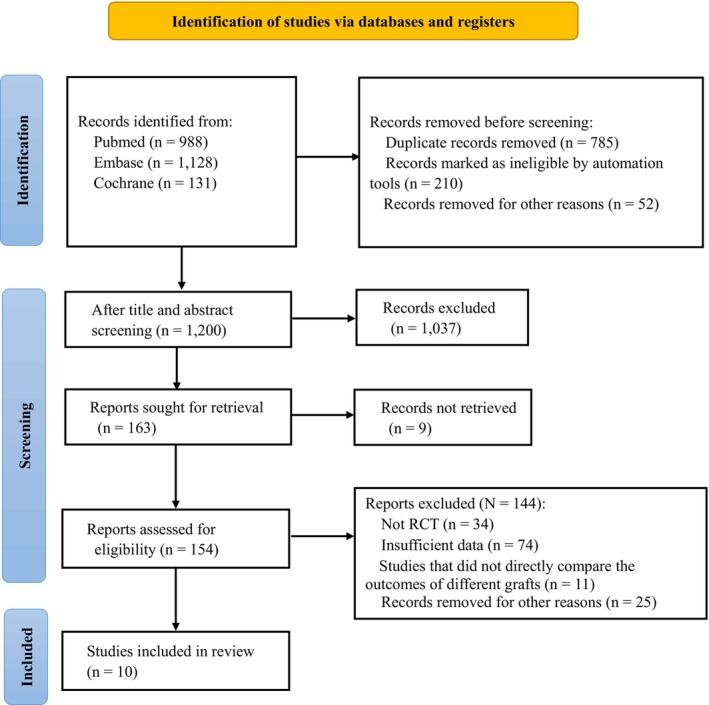
Flowchart of article selection process.

A total of 2247 kinds of literature were obtained by searching keywords, including PubMed (*n* = 988), Embase (*n* = 1128), and the Cochrane Library (*n* = 131). After layer‐by‐layer screening, 10 RCTs [14, 19, 30, 31, 32, 33, 34, 35, 36, 37] were ultimately included. Figure 1 illustrates the search strategy and screening process for this review.

### 3.2 Characteristics of the Included Studies

The 10 studies [14, 19, 30, 31, 32, 33, 34, 35, 36, 37] included in the meta‐analysis were all published in English and included a total of 668 patients, 338 patients in the experimental group (anterior cruciate ligament reconstruction with remnant preservation, group R), and 330 patients in the control group (standard anterior cruciate ligament reconstruction, group C). Postoperative IKDC scores were reported in five RCTs [30, 31, 32, 36, 37]; the Lysholm scores were reported in seven studies [19, 31, 32, 33, 34, 36, 37]; KT1000/2000 arthrometer side‐to‐side differences were reported in seven studies [14, 19, 32, 33, 34, 35, 36]; Lachman tests and pivot shift tests were each reported in four articles [19, 31, 36, 37] and five studies [19, 31, 32, 34, 36]. Postoperative proprioception evaluations were reported in two studies [14, 19]; grafts synovial coverage was reported in two RCTs [34, 36]; postoperative range of motion was reported in four studies [30, 31, 32, 36]; and the incidence of cyclops lesions was compared in five studies [19, 30, 31, 32, 36]. Table 1 summarizes the details of the studies included in this meta‐analysis.

**TABLE 1:** Distributions of factors related to final results within studies.

Author	Year	Treatment	Sample size	Mean age (years)	Sex (M, F)	Time from injury to surgery (mo)	Follow‐up period (mo)	Clinical outcomes
Gohil et al. [30]	2007	SB ACLR with remnant preservation	24	30.5	14, 10	2.55	12	IKDC, cyclops lesion, ROM
		Standard SB ACLR	25	35.5	13, 12	2.05	12	
Hong et al. [19]	2012	SB ACLR with remnant tensioning and suturing	45	34	33, 12	10.3	25.8	Lysholm, Lachman, pivot shift, SSD, synovial coverage, proprioception, cyclops lesion
		Standard SB ACLR	45	28	34, 11	9.4	35.5	
Demirağ et al. [31]	2012	SB with remnant preservation	20	31	18, 2	2.3	24	IKDC, Lysholm, Lachman, pivot shift, cyclops lesion, ROM
		Standard SB ACLR	20	28	18, 2	8	24	
Pujol et al. [32]	2012	SB ACLR with remnant augmentation	29	31.24	16, 13	5.32	12	IKDC, Lysholm, KOOS, pivot shift, SSD, cyclops lesion, ROM
		Standard SB ACLR	25	28.56	17, 8	4.6	12	
Zhang et al. [33]	2012	SB ACLR with remnant preservation	27	23.5	19, 4	12.7	24.4	Lysholm, SSD
		Standard SB ACLR	24	25.3	21, 5	10.2	25.2	
Lu et al. [34]	2015	DB ACLR with remnant preservation	36	29.3	36, 0	3.2 (weeks)	34.7	Lysholm, pivot shift, SSD, synovial coverage, ROM
		Standard DB ACLR	36	31.4	36, 0	3.2 (weeks)	39.6	
Andonovski et al. [35]	2017	SB ACLR with remnant preservation	33	26.6	NA	NA	7	SSD, proprioception
		Standard SB ACLR	33	26.4	NA	NA	7	
Kim et al. [36]	2020	SB ACLR with remnant tensioning	33	33.6	27, 6	1.5	28.7	IKDC, Lysholm, Lachman, pivot shift, SSD, synovial coverage, ROM
		Standard DB ACLR	34	29.1	28, 6	1.6	28.7	
El‐Desouky et al. [14]	2022	SB ACLR with remnant tensioning and suturing	56	27.7	52, 4	4.1	24	Lysholm, SSD, proprioception
		Standard SB ACLR	53	27.7	52, 1	3.8	24	
Wang et al. [37]	2022	SB/dB ACLR with remnant suturing	35	29.31	28, 7	NA	12	IKDC, Lysholm, Lachman
		Standard SB/dB ACLR	35	28.89	26, 9	NA	12	

Abbreviations: DB ACLR, double bundles anterior cruciate ligament reconstruction; F, female; IKDC, international knee documentation committee score; Lachman, Lachman test; Lysholm, Lysholm score; M, male; mo, month; NA, not available; pivot shift, pivot shift test; ROM, range of motion; SB ACLR, single bundle anterior cruciate ligament reconstruction; SSD, KT1000/2000 arthrometer side‐to‐side difference.

### 3.3 Quality Assessmentof the Selected Studies

**FIGURE 2:**
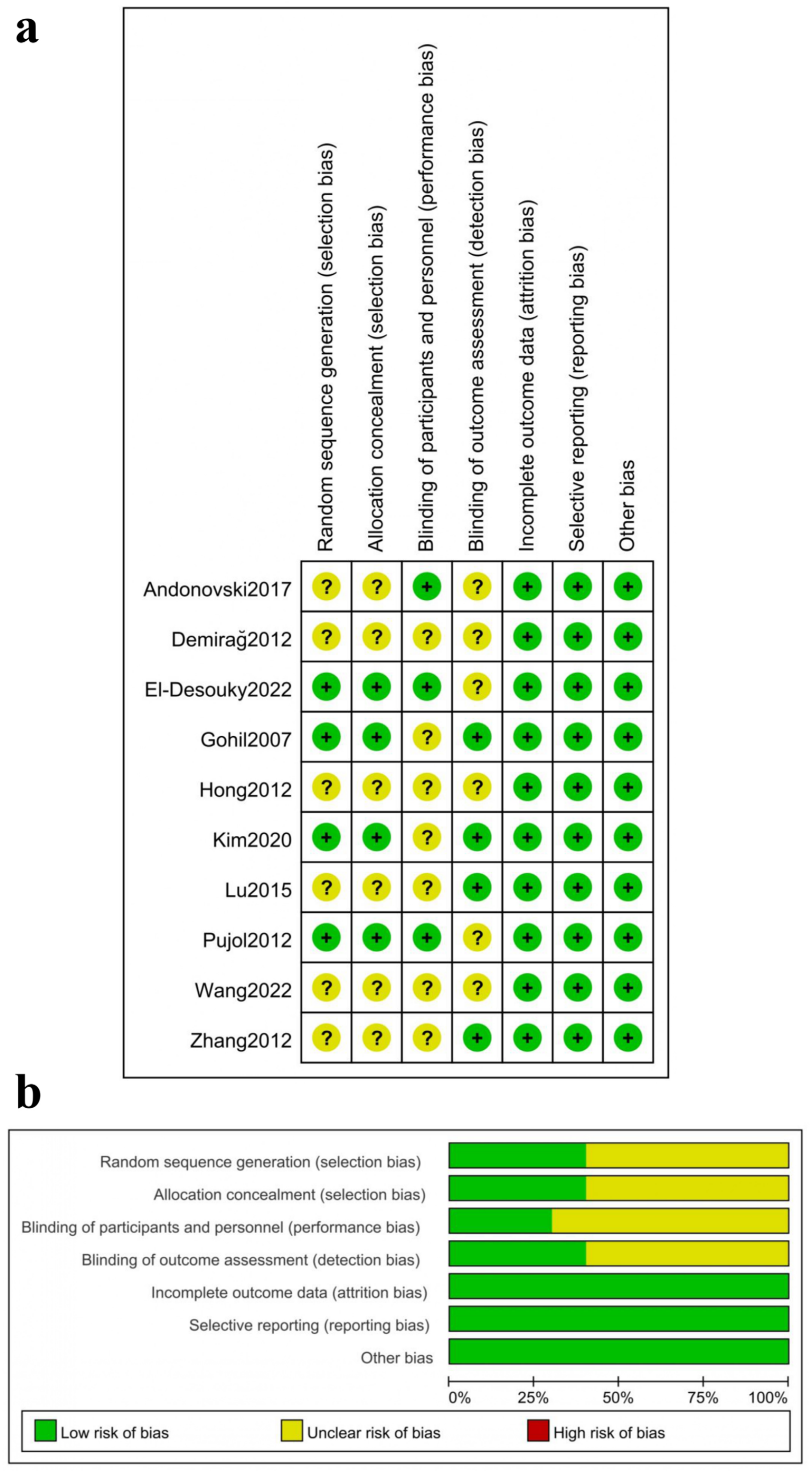
The risk of bias graph for each study (a) and summaries of risk of bias for the 10 randomized controlled trials (b).

Summaries of the risk of bias and the risk of bias graph for each study are presented in Figure 2. In general, the studies included were of a low risk of bias in terms of the incomplete outcome data, selective reporting, and other bias. However, some studies were found to have unclear risk of bias for the random sequence generation, allocation concealment, blinding of participants and personnel, as well as blinding of outcome assessment.

### 3.4 Evidence Certainty Evaluation of the Selected Studies

Table 2 shows the level of evidence certainty evaluation according to GRADE recommendations. The GRADE analysis indicated that the quality of evidence was relatively moderate for five outcomes (IKDC score, Lysholm score, Lachmant test, pivot shift test and cyclops lesions), relatively low for three outcomes (SSD, ROM, proprioception evaluation) and very low for synovial coverage.

**TABLE 2:** Level of evidence certainty evaluation according to GRADE recommendations.

Quality assessment							Quality	Importance
No of studies	Design	Risk of bias	Inconsistency	Indirectness	Imprecision	Other considerations		
IKDC 5	RCT	Serious	No serious inconsistency	No serious indirectness	No serious imprecision	None	MODERATE	CRITICAL
Lysholm 7	RCT	Serious	No serious inconsistency	No serious indirectness	No serious imprecision	None	MODERATE	CRITICAL
Lachman 4	RCT	Serious	No serious inconsistency	No serious indirectness	No serious imprecision	None	MODERATE	CRITICAL
Pivot shift 5	RCT	Serious	No serious inconsistency	No serious indirectness	No serious imprecision	None	MODERATE	CRITICAL
SSD 7	RCT	Serious	Serious	No serious indirectness	No serious imprecision	None	LOW	CRITICAL
Synovial coverage 2	RCT	Serious	Serious	No serious indirectness	Serious	None	VERY LOW	NOT IMPORTANT
Cyclops lesions 5	RCT	Serious	No serious inconsistency	No serious indirectness	No serious imprecision	None	MODERATE	NOT IMPORTANT
ROM 4	RCT	Serious	No serious inconsistency	No serious indirectness	Serious	None	LOW	NOT IMPORTANT
Proprioception 2	RCT	Serious	No serious inconsistency	No serious indirectness	Serious	None	LOW	NOT IMPORTANT

Abbreviations: IKDC, international knee documentation committee score; Lachman, Lachman test; Lysholm, Lysholm score; pivot shift, pivot shift test; RCT, randomized controlled trial; ROM, range of motion; SSD, KT1000/2000 arthrometer side‐to‐side difference.

### 3.5 Primary Outcomes

**FIGURE 3:**
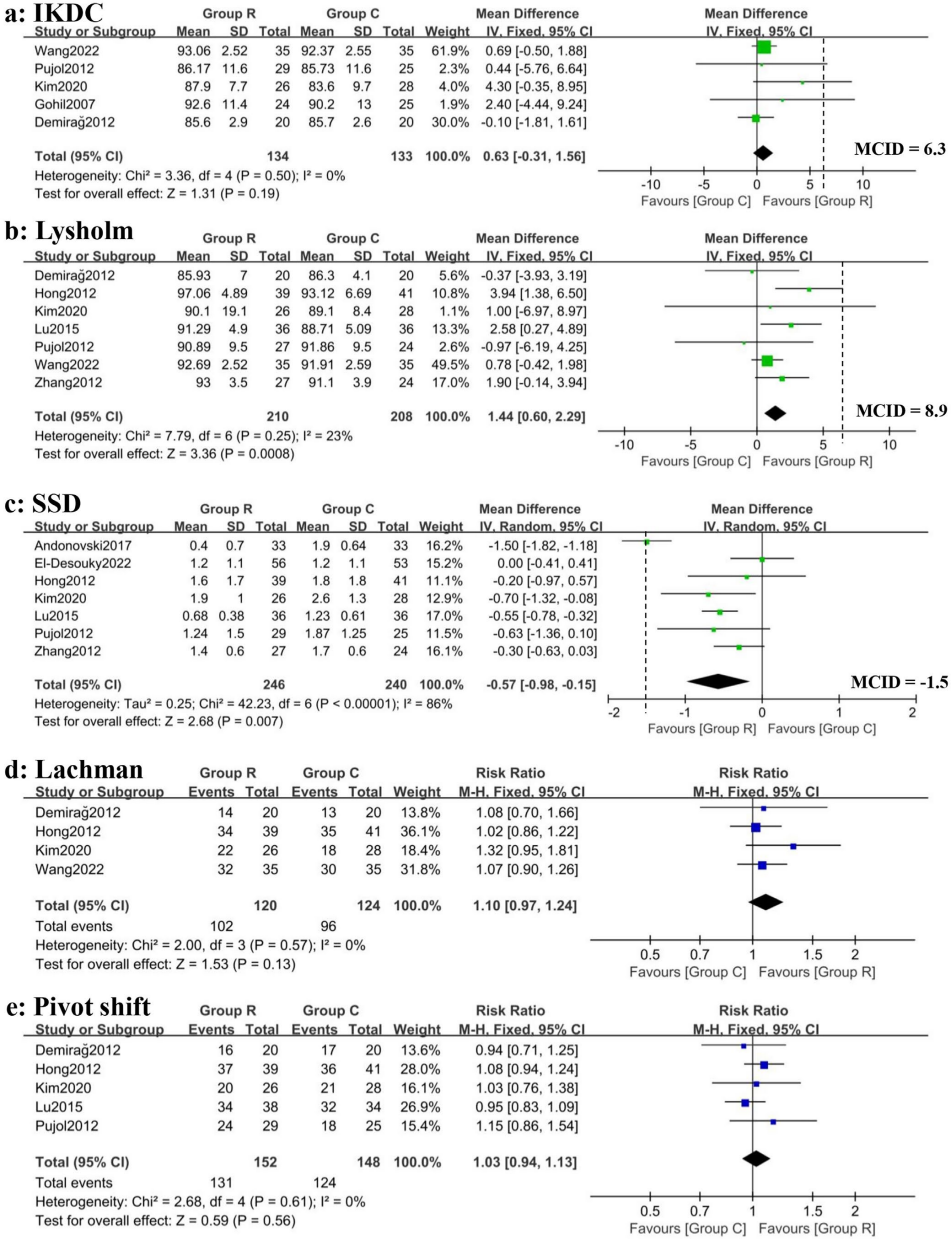
Forest plots for primary outcomes of included studies, including postoperative IKDC scores (a), Lysholm scores (b), KT‐1000/2000 side‐to‐side differences (c), Lachman tests (d) and pivot shift tests (e).

Statistically significant differences were found between groups R and C in favor of the remnant preservation technique, in terms of postoperative Lysholm scores (MD 1.44; 95% CI, 0.60–2.29; *I* ^2^ = 23%; *p* < 0.01) (GRADE: Moderate) and side‐to‐side difference (MD −0.57; 95% CI, −0.98 to −0.15; *I* ^2^ = 86%; *p* < 0.01) (GRADE: Low). There were no significant differences between the remnant preservation group and the standard technique group with respect to postoperative IKDC scores, Lachman tests, and the pivot shift tests. The results of the primary outcomes are illustrated in Figure 3 and Table 3.

**TABLE 3:** Summary of findings of primary outcomes.

Clinical outcomes	Importance	Anticipated absolute effects	MD/MCID%	GRADE evidence certainty	Interpretation of clinical relevance
IKDC	Critical	0.63 (−0.31, 1.56)	10%	Moderate	Clinically equivocal
Lysholm	Critical	1.44 (0.60, 2.29)	16%	Moderate	Clinically equivocal
SSD	Critical	−0.57 (−0.98, −0.15)	38%	Low	Clinically equivocal
Lachman	Important	1.10 (0.97, 1.24)	NA	Moderate	Clinically meaningless
Pivot shift	Important	1.03 (0.94, 1.13)	NA	Moderate	Clinically meaningless

*Note*: The data of anticipated absolute effects are presented as RR/MD (95% CIs).

Abbreviations: IKDC, international knee documentation committee score; Lachman, Lachman test; Lysholm, Lysholm score; MD/MCID%, the ratio of mean difference to minimal clinically important difference; NA, not available; Pivot shift, pivot shift test; SSD, KT1000/2000 arthrometer side‐to‐side difference.

### 3.6 Secondary Outcomes

There was a significant difference between the two groups concerning postoperative proprioception evaluation in favor of the remnant preservation group (MD −0.57; 95% CI, −0.83 to −0.31; *I* ^2^ = 0%; *p* < 0.01) (GRADE: Low). The results of the meta‐analysis showed no significant differences between groups R and C in terms of postoperative synovial coverage, cyclops lesions, and range of motion (Figure S1).

### 3.7 Clinical Interpretation of Key Outcomes

Although there was a statistically significant difference in Lysholm scores (*p* < 0.01), the MD was 1.44, which was far below the threshold of 8.9 previously reported to represent a clinically significant difference. Although the KT‐1000/2000 side‐to‐side difference showed statistical improvement, the 0.57 mm difference represents < 40% of the 1.5 mm threshold patients need to perceive stability changes. Despite the reported MD (−0.57°) being statistically significant in terms of postoperative proprioception recovery, this observed difference is so small that it is unlikely to be clinically relevant.

### 3.8 Publication Bias and Sensitivity Analysis

No asymmetry was observed in the funnel plots of nine different outcomes (Figure S2). Therefore, we concluded publication bias is unlikely to have substantially influenced the results of this meta‐analysis. We conducted sensitivity analyses utilizing the sequential elimination method in two outcomes (postoperative SSD, synovial coverage), due to their high heterogeneity. The results of sensitivity analyses showed no statistically significant change in the outcomes of the meta‐analysis once literature elimination had been implemented, thus suggesting that the results were both stable and reliable (Figure S3).

### 3.9 Subgroup Analysis

To explore potential modifiers of different follow‐up duration (≤ 12 months and ≥ 24 months) and remnant preservation techniques (tensioning or suturing and simple preservation), we conducted subgroup analyses for three outcomes failing to reach MCID thresholds (postoperative IKDC score, Lysholm score and SSD). Due to the small number of included studies, subgroup analysis for postoperative proprioception recovery was not feasible.

The mean difference between groups R and C did not show a statistically significant advantage in terms of clinical significance (not exceeding the MCID), regardless of whether the patients were evaluated in the short‐term postoperative period (≤ 12 months) or after a longer period (≥ 24 months). The subgroup analyses revealed that neither the suturing/tensioning remnant technique nor the relatively simple remnant‐preserving technique exhibited a clinically meaningful advantage over the traditional standard technique with respect to three clinical outcomes (Figure S4). The follow‐up duration and remnant preservation techniques did not emerge as the key factors influencing the difference between remnant preservation ACLR and standard ACLR, in terms of postoperative IKDC score, Lysholm score, and SSD.

## 4 Discussion

This systematic review and meta‐analysis demonstrated that there were no clinically meaningful differences between remnant‐preserving and standard ACLR techniques in terms of IKDC scores, Lysholm scores, side‐to‐side difference, and proprioception recovery. No differences were found in Lachman tests or pivot shift tests on meta‐analyses. In conclusion, given that there is no higher risk of complications and similar clinical outcomes to the standard technique, remnant preservation may be considered primarily when technical feasibility is high and remnant quality is optimal.

### 4.1 Primary Outcomes

The primary concern for patients undergoing ACLR is postoperative static and dynamic knee stability. The assessment of static stability is typically performed using measures such as Lachman test, pivot shift test, and SSD, whereas dynamic stability mainly depends on the recovery of proprioception. Theoretically, remnant preservation could accelerate graft maturation and enhance innervation, possibly leading to superior long‐term protection. However, though we found statistically significant differences in SSD and proprioception recovery, these differences were far below the threshold previously reported to represent a clinically significant difference. Interestingly, Andonovski et al. [35] found that, at the 6 months follow‐up period, there was a clinically significant difference between the remnant preservation group and the standard group in favor of the remnant‐preserving technique in terms of SSD (MD = −1.5 mm). The finding of this study and the subgroup analyses suggest that variations in SSD may only become apparent in the early postoperative period within the first 6 months, after which grafts from both techniques ultimately demonstrate adequate mechanical strength (≥ 12 months). Furthermore, the proprioceptive function of the knee joint is not exclusively dependent on the ACL, which is influenced by a variety of factors, including the muscles, menisci, ligaments, and skin [38, 39]. Recently, An et al. [40] evaluated brain activity changes in fully recovered ACL‐reconstructed patients compared with normal healthy individuals using electroencephalogram and found that ACLR patients had postural control patterns similar to those of healthy individuals but different brain activity patterns during early single‐leg stance. This finding indicates that proprioception involves afferent, central nerve system processing and motor activity in a closed loop, in which central processing is the main component. Therefore, for assessing proprioceptive recovery of patients with ACLR, it is recommended that measurements such as postural control on a moving platform be used instead of a passive movement test.

Beyond restoring knee stability, it is important to consider pain relief and postoperative mobility improvement for patients with ACLR. Some studies reported that remnant preservation ACLR had no apparent advantages in terms of IKDC or Lysholm score compared with standard technique [19, 41]. Though a prospective cohort study revealed that the Lysholm score, IKDC score, and KOOS at 6 months were greater in the synthetic remnant‐preserving group than in the hamstring autograft non‐preserving group [42], the initial strength of the synthetic material grafts may have contributed to the improved patient‐reported outcome measures in a short time, and the difference between the two groups might diminish with further revascularization of the autografts. Our meta‐analyses found no clinically important differences in the above measures with heterogeneity in graft sources (autografts, allografts and synthetic ligaments) among the included studies. Consequently, the interpretation of the overall pooled effect estimate should be approached with extreme caution. It represents an average across fundamentally different grafts. The favorable outcomes observed in certain studies using autografts may be mitigated by the less favorable outcomes observed in studies using allografts or synthetics, and vice versa. Furthermore, it is recommended that future clinical studies focus on maintaining consistency in grafts, rehabilitation protocols, and follow‐up times to explore the true effects of remnant preservation techniques.

### 4.2 Secondary Outcomes

Some studies on animals have suggested that preserved remnants might have benefits as biological supports to accelerate the process of revascularization and ligamentization of grafts and potentially help to preserve proprioception [8, 43, 44]. However, whether these benefits lead to important differences in outcomes in clinical studies is still being debated [13, 19, 33, 34, 41, 45, 46]. Our meta‐analysis showed the postoperative synovial coverage was similar in two groups. Moreover, the subgroup analyses revealed that neither the suturing/tensioning remnant technique nor the relatively simple remnant‐preserving technique exhibited a clinically meaningful advantage over the traditional standard technique. Interestingly, a cohort study demonstrated a significant correlation between laxity and the degree of initial graft coverage [13]. With the modified Crain classification, many remnant‐preserved reconstruction techniques can be rationally used to completely preserve the remnant ligament tissue during surgery and improve knee joint function and joint stability with few complication [37]. Given that remnant preservation techniques did not emerge as the key factors influencing the difference between remnant preservation ACLR and standard ACLR, exploring the true function of the ACL remnant, the potential impact of different initial remnant coverages on patient prognosis is worthy of attention.

Cyclops lesions are nodules or hypertrophied graft tissue commonly associated with a more anteriorly placed tibial tunnel or a disorganized mass of scar tissue in the anterior compartment, and they are the second most common cause of restricted knee range of motion after graft impingement [12, 45, 47]. One study reported that the incidence of cyclops syndrome was significantly greater in the remnant preservation group than in the non‐preservation group with double‐bundle ACL reconstruction using hamstring autografts [41]. This may be due to a greater graft volume in double‐bundle ACL reconstruction, increasing the risk of cyclops lesions. Tanabe et al. [48] reported no difference in the incidence of cyclops lesions between remnant‐preserving ACLR and remnant‐resecting ACLR. One MRI‐based study comparing standard and stump preservation ACLR techniques reported no difference in the incidence of cyclops lesions [49]. Similar to previous studies, our study did not find clear evidence of the differences between group R and group C in terms of the incidence of cyclops lesions and deficiency of ROM. This may be attributable to the fact that, for these two outcomes, the literatures included in the meta‐analyses exclusively encompassed single‐bundle ACL reconstructions. In comparison with grafts that contain double bundles, grafts comprising single bundles may exhibit reduced joint cavity contents, thereby diminishing the probability of impingement with ligament remnants.

Bone tunnel enlargement is an essential factor that affects the prognosis of ACLR patients [6, 7, 16, 50]. The mechanism of tunnel enlargement after ACL reconstruction is multifactorial, including various biological and mechanical factors, such as inflammatory and immune responses, quality of the bone, heat necrosis as a response to drilling, placement of the bone tunnel, the type of fixation, stress exerted by the graft, and an aggressive rehabilitation protocol. The influence of the residual ACL on bone tunnel enlargement remains debatable [51, 52]. Zhang et al. [33] discovered that preserving the remnant during ACL reconstruction may reduce the incidence of tunnel enlargement. However, it did not affect the short‐term clinical outcome of the procedure. Chen et al. [42] reported that there was a significant difference in bone tunnel widening between the two groups in the posteroanterior tibial, lateral tibial, posteroanterior femoral, and lateral femoral views, indicating less tunnel widening with remnant‐preserving ACLR. Multivariate logistic analysis by Koyama et al. [53] revealed that remnant preservation significantly reduced tunnel enlargement in the femoral AM, femoral PL, tibial AM, and tibial PL. However, few scholars have confirmed that there are no significant differences in the locations of the created tunnels or the incidence of tunnel coalition between the remnant‐resecting and remnant‐preserving procedures [54]. To promote graft‐to‐bone healing and avoid potential bone tunnel enlargement, Wan et al. [55] discovered a method of measuring intraoperative graft‐tunnel motion (GTM) and quantifying femoral tunnel position on postoperative 3D‐CT, which could guide clinicians to place the graft in a nearly isometric state and then minimize the GTM.

Graft rerupture is a significant cause of revision surgery in patients who have undergone ACLR. After primary ACLR, graft rupture may occur for various reasons, including delayed or inadequate revascularization of the new tissue [56]. ACLR with remnant preservation may reduce the risk of failure through improvement and acceleration of graft ligamentization and proprioceptive capacity [57]. A randomized controlled trial revealed similar re‐tear rates in both groups and earlier retears after remnant‐preserved ACLR (although underpowered for statistical comparison) [56]. In addition to graft ruptures, Crawford et al. [58] described the definition of “cumulative failure” which included clinical failures such as recurrent laxity, pathological ACL graft laxity, and ACL graft discontinuity. Chen et al. [42] reported that clinical failure occurred earlier in the synthetic with remnant‐preservation group than in the hamstring autograft with non‐preservation group. Due to their lack of viscoelasticity and increased stiffness compared to autologous tissue, synthetic grafts are more prone to influence the bone tunnel, resulting in inaccurate tunnel positioning, which may explain why clinical failure occurs earlier in patients with synthetic grafts than in those with natural grafts such as hamstring autografts. In contrast, Van Keulen et al. [59] showed that in ACLR surgery, the preservation of residual continuous but mechanically insufficient ACL tissue might lead to a lower clinical failure rate and ACL revision surgery rate than in standard ACLR. In summary, existing reviews have disagreed on which approach yields a better graft failure rate, and graft failure is dependent on various factors such as tendon‐bone healing, graft remodeling, and cumulative wear effect on grafts.

## 5 Limitations

This meta‐analysis has several limitations. Firstly, the number of RCTs included was relatively small; it was not feasible to quantitatively assess graft re‐injuries and bone tunnel enlargement. Furthermore, it may result in insufficient data with which to detect subtler significant differences and limit the power to detect bias. Clinicians should therefore be cautious about applying the results of this study in clinical practice. Secondly, a number of included studies exhibited an unclear risk of bias, particularly regarding blinding—a common limitation in surgical trials due to the difficulty of blinding surgeons. However, most postoperative assessments were performed by non‐surgical clinicians or rehabilitation specialists, reducing potential bias from unblinded surgeons. Nevertheless, assessors’ awareness of treatment allocation may have influenced subjective outcomes such as the Lachman and pivot‐shift tests. These findings should be interpreted with caution in clinical practice. Thirdly, though subgroup analyses were performed for follow‐up periods of less than 12 months and longer than 24 months, it is important to note that the longest follow‐up period of the included studies did not exceed 40 months. This explicitly cautions against extrapolating short‐term benefits to long‐term outcomes. Therefore, whether there is a difference between the two surgical approaches after longer periods of time requires further exploration. Finally, it is important to note that the confounding variables in each study include different preoperative remnant status, tunnel position, graft type, and graft size, which may have resulted in different postoperative outcomes. Further research is necessary to compare these technologies, and consistency in assessment methods is also crucial to ensure reliable results.

## 6 Conclusion

Given moderate‐certainty evidence showing no clinically meaningful benefit in postoperative knee stability or function, remnant preservation may be considered primarily when technical feasibility is high and remnant quality is optimal. This is achieved without increasing the risk of cyclops lesions and deficiency of ROM. However, further studies are needed to support its clinical superiority, focusing on quantitatively measuring bone tunnel enlargement and graft re‐injuries.

## Author Contributions

**Chao Fan Chen:** conceptualization, data curation, investigation, validation, formal analysis, visualization, project administration, resources, writing – original draft, writing – review and editing, software. **Ling Yao Kong:** data curation, formal analysis, investigation, project administration. **Tao Li:** investigation, data curation, formal analysis. **Lei Yao:** formal analysis, data curation. **Yang Xu:** formal analysis, data curation. **Li Wang:** formal analysis, data curation. **Hong Yu Zhou:** formal analysis, data curation. **Jian Li:** conceptualization, funding acquisition, data curation, methodology, writing – review and editing, supervision.

## Disclosure

The authors have nothing to report.

## Conflicts of Interest

The authors declare no conflicts of interest.

## Supporting information


**Figure S1:** Forest plots for secondary outcomes of included studies, including postoperative synovial coverage (a), proprioception recovery (b), range of motion (c), and cyclops lesions (d).

**Figure S2:** Funnel plots for nine different outcomes of included studies, including postoperative IKDC scores (a), Lysholm scores (b), Lachman tests (c), KT‐1000/2000 side‐to‐side differences (d), pivot shift tests (e), synovial coverage (f), proprioception recovery (g), cyclops lesions (h), and range of motion (i).

**Figure S3:** Sensitivity analyses for two outcomes of included studies, including postoperative KT‐1000/2000 side‐to‐side differences (a) and synovial coverage (b).

**Figure S4:** Subgroup analyses for three outcomes failing to reach MCID thresholds, including postoperative IKDC scores (a), Lysholm scores (b), and KT‐1000/2000 side‐to‐side differences (c).

**Data S1:** PRISMA_2020_checklist.

## Data Availability

The datasets analyzed during the current study are available in public articles.

## Appendix A Search History of PubMed

**Table:**

Search number	Query	Search details	Results
8	((Anterior Cruciate Ligament[MeSH Terms]) OR (Anterior Cruciate Ligament*[Title/Abstract])) AND (((remnant*[Title/Abstract]) OR (stump*[Title/Abstract])) OR (residual[Title/Abstract]))	(“anterior cruciate ligament”[MeSH Terms] OR “anterior cruciate ligament*”[Title/Abstract]) AND (“remnant*”[Title/Abstract] OR “stump*”[Title/Abstract] OR “residual”[Title/Abstract])	988
7	((remnant*[Title/Abstract]) OR (stump*[Title/Abstract])) OR (residual[Title/Abstract])	“remnant*”[Title/Abstract] OR “stump*”[Title/Abstract] OR “residual”[Title/Abstract]	306,781
6	residual[Title/Abstract]	“residual”[Title/Abstract]	253,308
5	stump*[Title/Abstract]	“stump*”[Title/Abstract]	19,746
4	remnant*[Title/Abstract]	“remnant*”[Title/Abstract]	37,431
3	(Anterior Cruciate Ligament[MeSH Terms]) OR (Anterior Cruciate Ligament*[Title/Abstract])	“anterior cruciate ligament”[MeSH Terms] OR “anterior cruciate ligament*”[Title/Abstract]	28,702
2	Anterior Cruciate Ligament*[Title/Abstract]	“anterior cruciate ligament*”[Title/Abstract]	26,243
1	Anterior Cruciate Ligament[MeSH Terms]	“anterior cruciate ligament”[MeSH Terms]	12,833

## Appendix B Search History of Embase

**Table:**

Session results			
No.	Query results	Results	Date
#8.	#6 AND #7	1,118	1 Nov 2024
#7.	#3 OR #4 OR #5	423,401	1 Nov 2024
#6.	#1 OR #2	33,485	1 Nov 2024
#5.	residual:ab,ti	353,837	1 Nov 2024
#4.	stump*:ab,ti	25,617	1 Nov 2024
#3.	remnant*:ab,ti	49,170	1 Nov 2024
#2.	‘anterior cruciate ligament*’:ab,ti	30,227	1 Nov 2024
#1.	‘anterior cruciate ligament’/exp	14,723	1 Nov 2024

## Appendix C Search History of Cochrane Library

Date Run: 08/11/2024 08:33:16

**Table:**

ID	Search	Hits
#1	MeSH descriptor: [Anterior Cruciate Ligament] explode all trees	853
#2	(Anterior Cruciate Ligament*):ti,ab,kw	3834
#3	(remnant*):ti,ab,kw	1842
#4	(stump*):ti,ab,kw	915
#5	(residual):ti,ab,kw	25,933
#6	#1 or #2	3834
#7	#3 or #4 or #5	28,396
#8	#6 and #7	131
