# Supplementary figures and images for: Remnant Preservation in Anterior Cruciate Ligament Reconstruction Versus Non‐Preservation Methods: A Systematic Review and Meta‐Analysis
Source: Orthop Surg. 2025 Sep 4;17(11):3022–35. doi: 10.1111/os.70167 (PMC12580229; doi:10.1111/os.70167)

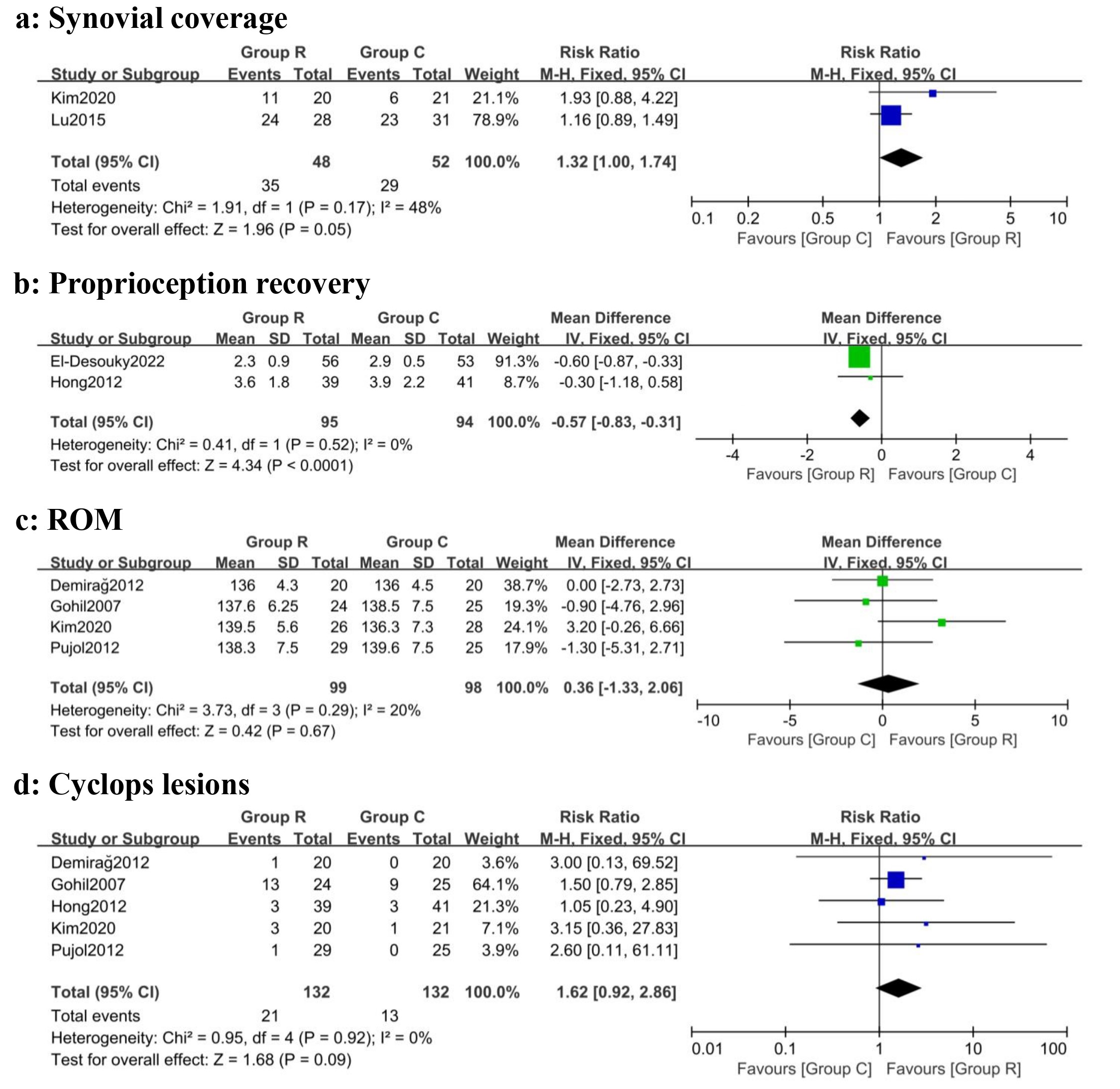

Supplement: Supplementary file 1 — Figure S1: Forest plots for secondary outcomes of included studies, including postoperative synovial coverage (a), proprioception recovery (b), range of motion (c), and cyclops lesions (d). [file OS-17-3022-s002.jpg]

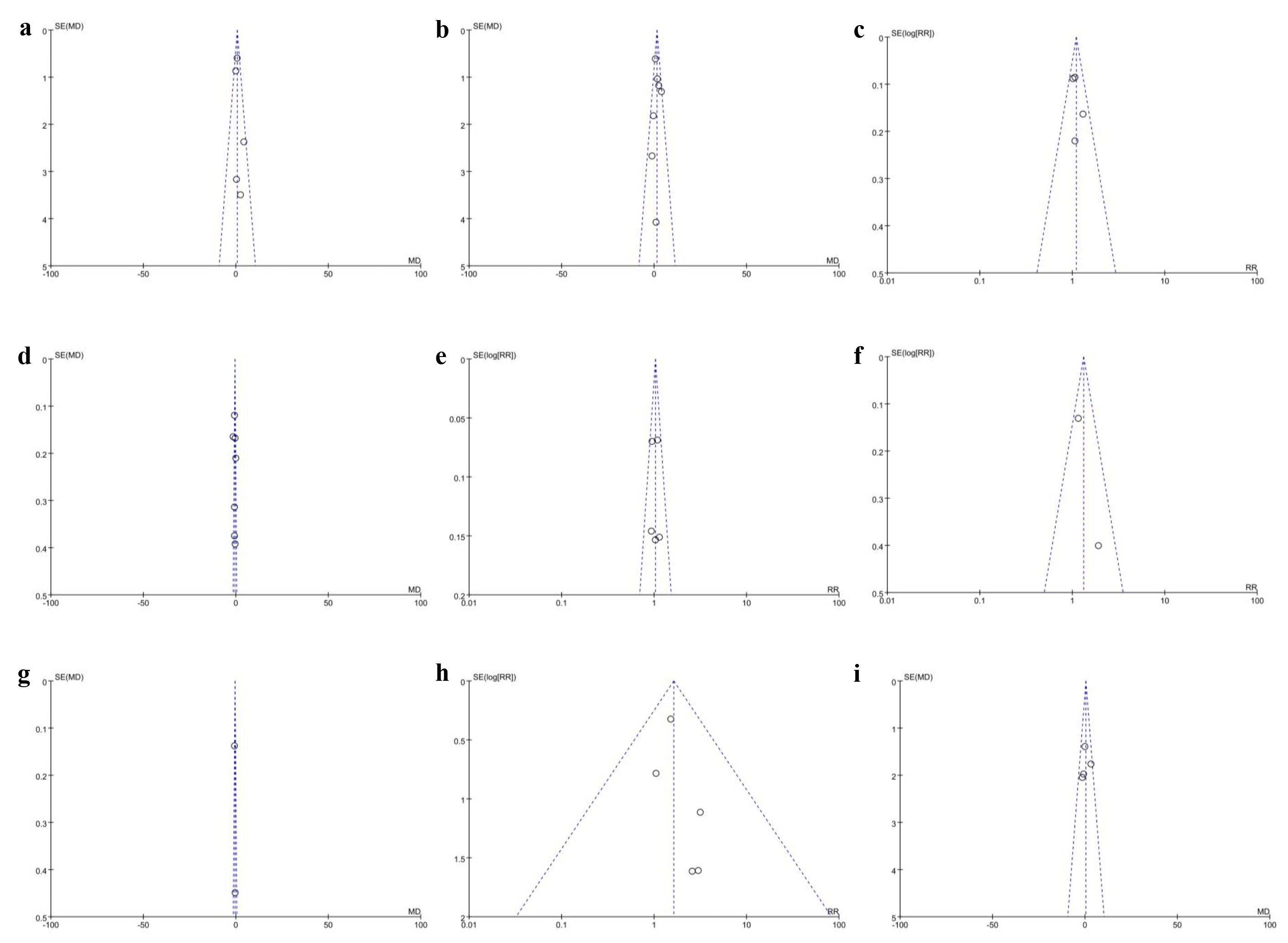

Supplement: Supplementary file 2 — Figure S2: Funnel plots for nine different outcomes of included studies, including postoperative IKDC scores (a), Lysholm scores (b), Lachman tests (c), KT‐1000/2000 side‐to‐side differences (d), pivot shift tests (e), synovial coverage (f), proprioception recovery (g), cyclops lesions (h), and range of motion (i). [file OS-17-3022-s001.jpg]

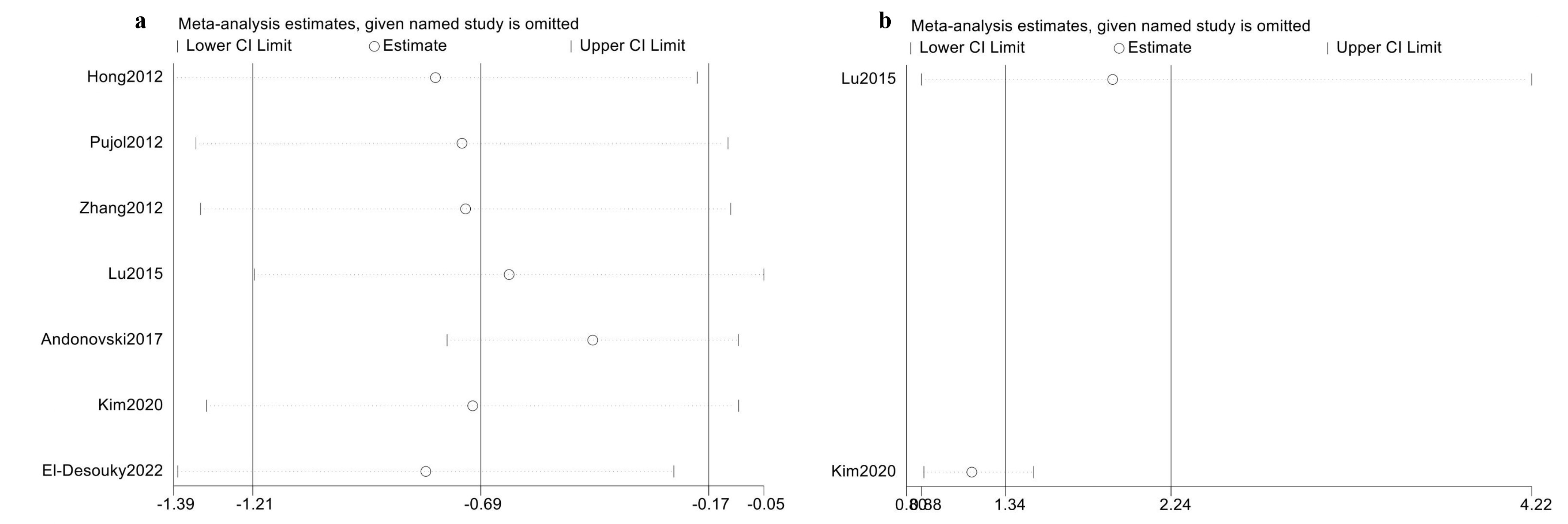

Supplement: Supplementary file 3 — Figure S3: Sensitivity analyses for two outcomes of included studies, including postoperative KT‐1000/2000 side‐to‐side differences (a) and synovial coverage (b). [file OS-17-3022-s005.jpg]

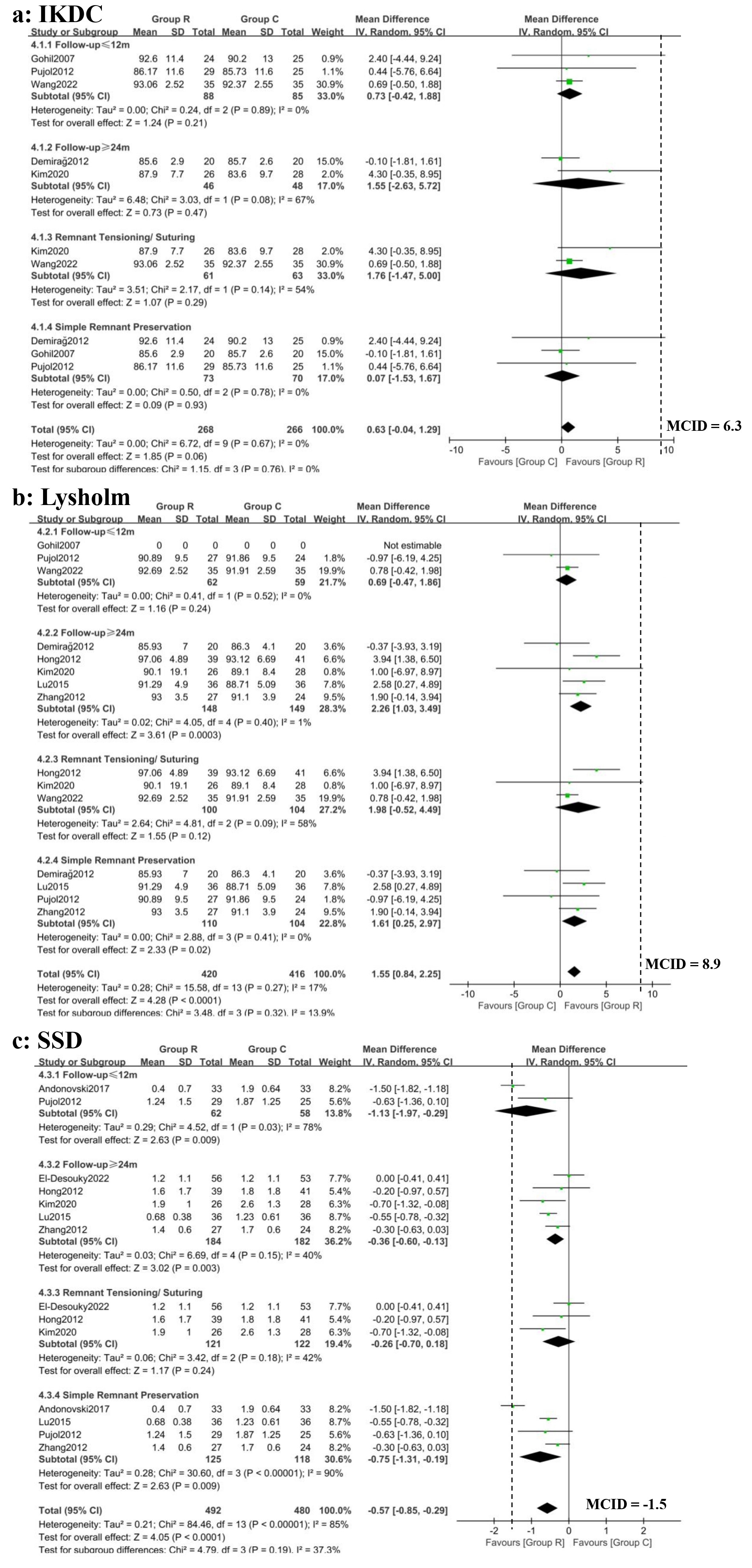

Supplement: Supplementary file 4 — Figure S4: Subgroup analyses for three outcomes failing to reach MCID thresholds, including postoperative IKDC scores (a), Lysholm scores (b), and KT‐1000/2000 side‐to‐side differences (c). [file OS-17-3022-s004.jpg]
